# Torque Teno Virus as a Biomarker for Infection Risk in Kidney Transplant Recipients: A Machine Learning-Enabled Cohort Study

**DOI:** 10.3390/idr17050107

**Published:** 2025-09-02

**Authors:** Sara Querido, Luís Ramalhete, Perpétua Gomes, André Weigert

**Affiliations:** 1 Renal Transplantation Unit, Nephrology Department, Hospital de Santa Cruz, Unidade Local de Saúde de Lisboa Ocidental, 2790-134 Carnaxide, Portugal; alweigert@gmail.com; 2 CHRC, NOVA Medical School, Faculdade de Ciências Médicas, NMS, FCM, Universidade NOVA de Lisboa, 1169-056 Lisboa, Portugal; 3 IPST—Instituto Português do Sangue e da Transplantação, Alameda das Linhas de Torres 117, 1769-001 Lisboa, Portugal; 4 NOVA Medical School, Faculdade de Ciências Médicas, NMS, FCM, Universidade NOVA de Lisboa, 1169-056 Lisboa, Portugal; 5 iNOVA4Health—Advancing Precision Medicine, Núcleo de Investigação em Doenças Renais, NMS—NOVA Medical School, FCM—Faculdade de Ciências Médicas, Universidade NOVA de Lisboa, 1169-056 Lisboa, Portugal; 6 Laboratory of Clinical Microbiology and Molecular Biology, Department of Clinical Pathology, Unidade Local de Saúde de Lisboa Ocidental, 1349-019 Lisboa, Portugal; gomes.perpetua@gmail.com; 7 Centro de Investigação Interdisciplinar Egas Moniz (CiiEM), IUEM, 2829-511 Almada, Portugal; 8 Pharmacology and Neurosciences Institute, Faculdade de Medicina, Universidade de Lisboa, 1649-004 Lisboa, Portugal

**Keywords:** TTV, kidney transplantation, machine learning, infection, immunosuppression

## Abstract

Background: Torque Teno Virus (TTV) viremia has been proposed as a marker for infection risk in kidney transplant (KT) recipients. This study aimed to evaluate the prognostic value of TTV levels for predicting infections post-KT. Methods: A cohort of 82 KT patients was analyzed. TTV loads were measured before KT and at the time of cutoff analysis (mean time since KT: 20.2 ± 10.3 months). Infections were tracked within six months following the time of cutoff analysis. Univariable analyses and a supervised machine learning approach (logistic regression with leave-one-out cross-validation) were conducted to rigorously assess TTV’s predictive ability for post-transplant infection. Results: Seventy-two patients (87.8%) had detectable TTV before KT. Of these, 30.5% developed infections, predominantly viral. TTV loads increased significantly from 3.35 ± 1.67 log_10_ cp/mL before KT to 4.53 ± 1.93 log_10_ cp/mL at the time of cutoff analysis. Infected patients had significantly higher TTV loads (5.39 ± 1.68 log_10_ vs. 4.16 ± 1.94 log_10_ cp/mL, *p* = 0.0057). The optimal TTV threshold for predicting infection at the time of cutoff analysis was 5.16 log_10_ cp/mL, with 60% sensitivity and 81% specificity. Machine learning models improved performance, with sensitivity and specificity 0.805 and 0.735, respectively. Conclusions: TTV viremia may serve as a biomarker for infection risk, particularly when used with other clinical variables. The identified TTV threshold of 5.16 log_10_ cp/mL offers a practical tool for clinical decision-making, particularly when integrated with a machine learning model. Further studies with larger cohorts are needed to validate these findings and refine clinical applications.

## 1. Introduction

Kidney transplantation (KT) is the best treatment for eligible patients with end-stage renal disease. Advances in immunosuppressive therapy for KT have reduced the incidence of graft rejection but have also increased the risk of infections and virally mediated malignancies [1].

Unfortunately, to date, no reliable biomarker has been established to assess the immune function of KT recipients. Clinically, the routine dosing of immunosuppressive drugs is primarily guided by the quantification of calcineurin or mTOR inhibitor trough levels in peripheral blood, which correlates more closely with the risk of drug-related toxicity than with the effectiveness of immunosuppression [2].

In this context, monitoring the peripheral blood levels of Torque Teno Virus (TTV) may represent a promising strategy. The discovery of TTV in 1997, followed by the identification of other members of the Anelloviridae family, introduced the concept of “commensal viruses,” which are considered normal constituents of the human virome and are not known to cause pathology in humans [3].

Although TTV can be detected in up to 90% of healthy individuals and has not been linked to any specific disease, recent studies have explored whether peripheral blood levels of TTV could reflect the overall strength of both innate and specific immunity [4]. Nonetheless, the precise biological mechanisms remain incompletely understood. It is hypothesized that TTV replication is modulated by T cell-mediated immune activity, but factors such as co-infections, inflammation, or viral genotype variation may also influence viral dynamics [5]. Additional data suggest TTV dynamics may also reflect immune activation or inflammation, not solely immunosuppression [6].

In clinical practice, the TTV viral load might potentially be used to predict increased risks of both rejection and infection, and, thus, it could be a tool to customize immunosuppression. Therefore, the quantification of the TTV viral load and/or TTV kinetics post-KT could serve as a predictive biomarker for the risk of rejection and infection in solid-organ transplant recipients. Previous studies have shown that low TTV levels are associated with graft rejection, while higher TTV levels correlate with an increased risk of infection. However, the ideal threshold for reducing immunosuppression and the optimal time points for measuring TTV viremia to guide immunosuppression modulation remain to be determined [4,7,8].

This study aims to assess the prognostic value of TTV levels in the development of clinical infections in a cohort of 82 prevalent KT patients. Given the complex interplay of immunological and clinical factors influencing infection risk, advanced analytic approaches such as machine learning (ML) may enhance the predictive value of TTV and support individualized patient management.

Machine learning (ML) is increasingly used in biomedical research to identify complex, non-linear patterns in multidimensional data that may not be captured by conventional statistical methods. In transplantation medicine, ML has shown promise in predicting graft outcomes, infections, and rejection episodes by integrating clinical, laboratory, and biomarker data. In this context, ML-based models can leverage the multifactorial nature of the immune response and infection risk, enabling more refined and individualized risk stratification. This study incorporates ML to evaluate the added predictive value of TTV viremia, potentially overcoming limitations inherent to single-variable or threshold-based analyses.

This study leverages both conventional statistics and ML to evaluate the prognostic value of TTV levels for the infection risk in KT recipients. Correlating TTV loads with clinical outcomes supports the potential role of TTV as a biomarker of functional immunity.

## 2. Materials and Methods

### 2.1. Study Design and Population

In this single-center retrospective cohort study, a total of 121 kidney transplant (KT) recipients were included. These recipients underwent KT between May 2019 and October 2022 at a Kidney Transplant Unit in Portugal, with a minimum follow-up of 3 months post-transplantation.

Scheduled clinical and laboratory evaluations were conducted immediately before KT and at predefined time points following transplantation. All participants provided informed consent. TTV viral load was measured at baseline and again at a second time point, which ranged from 3 to 40 months post-KT.

Patients with an undetectable TTV viral load (<100 copies/mL) at both baseline and at the time of cohort analysis (n = 26), as well as those with incomplete infection data outside the 3–40 months post-KT range (n = 21), were excluded. This resulted in a final cohort of 82 patients.

To enhance interpretability in a heterogeneous cohort, we explicitly characterized the transplant type and immunosuppression. Most recipients received kidneys from deceased donors, 71/82 (86.6%), with the remainder from living donors. Induction therapy included thymoglobulin in 52/82 (63.4%), and maintenance regimens combined an mTOR inhibitor with a calcineurin inhibitor in 26/82 (31.7%). These variables were screened as candidate covariates; they did not materially change the associations of interest and were not retained in the final parsimonious model to minimize overfitting in a small dataset.

### 2.2. Data Collection

Patients who developed infections within 6 months after the time of cutoff analysis were compared to those without infections in terms of age, gender, time elapsed since KT, estimated glomerular filtration rate (eGFR), induction and maintenance immunosuppression, diagnosis of delayed graft function, diabetes, type of previous dialysis, absolute TTV viral load at the time of cutoff analysis, and the TTV ratio (defined as the TTV viral load before KT divided by the absolute TTV viral load at the time of cutoff analysis).

Infectious events were defined as any bacterial, fungal, or viral infection that required antimicrobial or antiviral therapy or a reduction in immunosuppression.

Polyomavirus infections were defined according to the recommendations of the Banff Working Group and the American Society of Transplantation Infectious Diseases Community of Practice guidelines [9,10]. Briefly, a plasma BKPyV viral load ≥ 1 × 10^4^ copies/milliliter (cp/mL) was considered presumptive polyomavirus nephropathy (pPVAN), and polyomavirus nephropathy (PVAN) was confirmed by biopsy. Cytomegalovirus (CMV) viremia was defined by CMV replication in plasma.

Biopsy-proven acute rejection episodes were classified according to the 2019 update of the Banff classification [11].

This study adhered to the Declaration of Helsinki, followed national and international guidelines for health data protection, and was approved by the Ethics Committee of the “Centro Hospitalar de Lisboa Ocidental” (approval number 20170700050, date 22 July 2022).

The eGFR was calculated using the Chronic Kidney Disease Epidemiology Collaboration (CKD-EPI) equation [12].

The primary endpoint was the development of an infection within 6 months after TTV cohort analysis.

### 2.3. TTV Analysis

Quantitative TTV DNA viral load extraction from plasma samples and amplification of DNA were performed as previously described [13,14]. In brief, DNA extraction was carried out using the eMAG System (BioMerieux, Marcy, France). For DNA amplification and quantification, the Argene R-Gene TTV quantification kit (BioMerieux) was used on an Applied Biosystems 7500 (Thermofisher, Waltham, MA, USA) according to the manufacturer’s instructions. The R-Gene assay is designed to detect the majority of TTV genotypes (1, 6, 8, 10, 12, 15, 16, 19, 27, 28). The threshold defining positivity was 100 copies/mL, as defined by the manufacturer. Results are expressed in log_10_ copies/mL.

### 2.4. BKPyV and JCPyV Analysis

The presence of BKPyV and JCPyV were assessed in urine (viruria) and in plasma samples (viremia). For the detection of JCPyV and BKPyV, the commercial assays JCPyV ELITe MGB^®^ Kit (ELITechGroup SAS, Puteaux, France) and BKPyV ELITe MGB^®^ Kit (ELITechGroup SAS, Puteaux, France) were used. These assays are CE-IVD-validated on a diverse range of sample types, in combination with ELITe InGenius^®^ (ELITechGroup SAS, Puteaux, France), a fully automated sample-to-result solution. The primers and the JCPyV- and BKPyV-specific probe (stabilized by MGB^®^ group, labeled by FAM fluorophore, and quenched by a non-fluorescent molecule) are specific for the Large T antigen region of the JCPyV gene and the Large T antigen gene of BKPyV. The primers and the probe for the internal control (stabilized with MGB^®^ group, labeled by AP525 fluorophore, analogous to VIC, and quenched by a non-fluorescent molecule) are specific to the artificial DNA sequence.

The sample volume used to extract DNA was 200 µL. In both assays, two amplification reactions were performed starting from extracted DNA. For BKPyV, a specific primer for the region of the Large T antigen gene of BKPyV and a specific primer for the region of the human beta globin gene (internal control) were used; for JCPyV, a specific primer for the Large T antigen region of the JCPyV gene and a specific primer for an artificial sequence of DNA (internal control) were used. BKV- and JCPyV-specific probes with ELITE MGB^®^ technology, labeled with FAM fluorophore, are activated when they hybridize with the specific product of the BKPyV and JCPyV amplification reaction. The viral load is obtained, in each case, through a calibration curve. The threshold defining positivity for BKPyV was 165 copies/mL in plasma and 89 copies/mL in urine. For JCPyV, both plasma and urine thresholds were 500 copies/mL. Results are expressed in log_10_ copies/mL.

### 2.5. CMV Analysis

CMV viremia was assessed in plasma samples. Briefly, after DNA extraction, two amplification reactions are performed: a specific reaction for the exon 4 region of the CMV MIEA gene (major immediate early antigen, HCMVUL123) and a specific reaction for a region of the human beta globin gene (internal control of inhibition). The CMV-specific probe with ELITe MGB^®^ technology, labeled with FAM fluorophore, is activated when it hybridizes with the specific product of the CMV amplification reaction. The internal control-specific probe with ELITe MGB^®^ technology, labeled with AP525 fluorophore (analogous to VIC), is activated when it hybridizes with the specific product of the internal control amplification reaction. The viral load is obtained through a calibration curve. The threshold defining positivity was 178 copies/mL. Results are expressed in log_10_ copies/mL.

### 2.6. Immunosuppressive Protocols

All patients received triple maintenance immunosuppression, primarily consisting of tacrolimus, mycophenolic acid (MPA), and prednisolone, or tacrolimus, an mTOR inhibitor, and prednisolone. Tacrolimus was administered orally at a dose of 0.15 mg/kg/day, divided into two doses, and adjusted to maintain a target trough concentration between 4 and 10 ng/mL, depending on the time elapsed after KT. Prednisolone was prescribed starting on the fifth day after KT at a dose of 0.6 mg/kg and was tapered to 5 mg/day over the first 3 months post-KT. MPA (mycophenolate mofetil 1000 mg orally twice daily) was initiated after KT and reduced in the event of adverse effects. The daily dose was then adjusted to 1000–1500 mg after 3–6 months. Everolimus was started at 2.0 mg/day, with dose adjustments made to maintain target trough concentrations between 3 and 8 ng/mL.

### 2.7. Prophylaxis Protocols

All KT recipients received prophylaxis for *Pneumocystis jirovecii* pneumonia with either trimethoprim/sulfamethoxazole (480 mg daily) or atovaquone (750 mg twice daily) for 1 year. Valganciclovir (900 mg daily, adjusted according to kidney function) was administered for a duration of 6 months to patients whose induction therapy included antithymocyte globulin and/or rituximab, or to those in CMV IgG-negative recipients/CMV IgG-positive donor pairs.

### 2.8. Statistical Analysis

Baseline clinical and demographic data were analyzed using appropriate statistical tests. Continuous variables were first assessed for normality (e.g., via Shapiro–Wilk test); those following an approximately normal distribution were compared between groups using Student’s *t*-test, whereas non-normally distributed continuous variables were compared using the Mann–Whitney U test. Categorical variables were analyzed with chi-square tests. Statistical significance was defined at a two-tailed *p* < 0.05., using GraphPad Prism version 8.0.2 for Microsoft Windows (GraphPad Software, San Diego, CA, USA).

#### 2.8.1. Conventional Statistical Approach

The initial approach for establishing an optimal TTV viral load threshold to predict post-transplant infection relied on conventional diagnostic performance analysis. Receiver operating characteristic (ROC) curves were generated to assess the discriminative ability of the TTV viral load in identifying patients at an increased risk of clinically significant infections. Key metrics, including sensitivity, specificity, and the area under the ROC curve (AUC), were calculated to quantify model performance. To determine the most informative cutoff point, Youden’s Index (J = Sensitivity + Specificity − 1) was applied, as it identifies the threshold that optimally balances true positive and false positive rates, thereby maximizing overall diagnostic accuracy [15].

To further evaluate the robustness of this cutoff, subgroup analyses were conducted according to relevant clinical and demographic strata—such as age, sex, type of immunosuppressive induction, and time from transplantation to sampling. This ensured that the selected threshold retained discriminative power across heterogeneous patient profiles and was not driven by confounding or outlier subgroups.

In addition to these conventional analyses, we employed advanced data mining techniques (unsupervised and supervised learning) to explore complex patterns in the dataset, as detailed in Section 2.8.2 and Section 2.8.3. All machine learning and multivariate analyses were implemented in the Orange: Data Mining Toolbox, version 3.36.2 (Bioinformatics Lab, University of Ljubljana, Ljubljana, Slovenia), a visual programming toolkit for exploratory data analysis and predictive modeling [16].

Because sampling occurred at variable times after transplantation (3–40 months), temporal heterogeneity was addressed in two ways: (i) “time since transplantation (months)” was modeled as a continuous covariate; and (ii) an exploratory time-stratified analysis used the reviewer-requested windows <12, 12–24, and >24 months. Diagnostic metrics are reported with 95% confidence intervals (CIs): proportions (e.g., sensitivity, specificity) by Wilson score CIs, and AUC CIs by the Hanley–McNeil approximation for finite samples.

#### 2.8.2. Dimensionality Reduction and Unsupervised Visualization with t-SNE

To explore potential intrinsic patterns in the dataset and assess whether patients formed natural groupings based on their post-transplant profiles, we applied t-distributed Stochastic Neighbor Embedding (t-SNE). This non-linear dimensionality reduction method enables high-dimensional data to be projected onto a low-dimensional space, typically two dimensions, while preserving the relative distances between samples in the original feature space [17]. It is particularly useful for visualizing hidden structures in complex clinical datasets where standard bivariate techniques may fail to reveal associations.

In our analysis, we considered the TTV load, demographic variables (e.g., age, sex), transplant-specific data (e.g., induction therapy, prior rejection), and key laboratory markers (e.g., lymphocyte count, tacrolimus trough levels).

The t-SNE embedding was generated, with the “preserve global structure” mode enabled. This configuration leverages a dual-perplexity scheme (combining local and global views) to simultaneously capture fine-grained clustering and broader spatial relationships between distinct patient subsets. This is especially relevant in transplant populations, where patients may differ subtly in immunological or virological parameters, but still share broader clinical trajectories.

The output was a two-dimensional scatterplot in which each point represented an individual patient, positioned such that proximity reflected multivariate similarity. We examined these maps to identify whether patients clustered according to post-transplant infection status, allowing for qualitative insights into whether biomarker and clinical profiles were associated with outcomes. Clusters where infected and non-infected individuals appeared well separated were taken as exploratory evidence of underlying discriminative structure in the dataset.

While t-SNE does not produce predictive models, it supports hypothesis generation and complements subsequent supervised analyses by visually illustrating data heterogeneity. In the context of a limited sample size, it also provides a valuable sanity check before proceeding to more formal classification efforts.

#### 2.8.3. Supervised Classification, Feature Extraction, and Nomogram Interpretation

In addition to unsupervised exploration, we applied a supervised learning framework centered on logistic regression (LR), selected for its transparency, interpretability, and stability in small clinical datasets. Unlike black-box models, LR provides direct insights into the influence of each feature on the predicted outcome, which is particularly important when clinical interpretability is prioritized over pure predictive performance [18].

Given the moderate sample size and retrospective nature of this study, we employed leave-one-out cross-validation (LOOCV) to evaluate model generalizability. In this strategy, each sample is used once as the test set while the remainder of the dataset forms the training set, iteratively cycling through all samples. LOOCV is particularly well suited to small cohorts, as it maximizes the use of available data and ensures that the model is evaluated on truly independent samples in each iteration, thereby reducing overfitting and providing a nearly unbiased estimate of predictive performance [19,20].

To reduce dimensionality and enhance model performance, feature extraction was performed using the information gain (IG) criterion, an entropy-based univariate metric that quantifies the contribution of each feature to class discrimination. IG evaluates how much information a given feature provides about the target class by measuring the reduction in entropy when the feature is known. Features with higher IG scores were retained for model development, while those with negligible scores were discarded to mitigate noise and overfitting risks [21].

After feature extraction, the final LR model was constructed, incorporating the most informative features. Performance metrics, such as sensitivity, specificity, accuracy, and area under the ROC curve, were calculated by aggregating predictions across all folds.

To enhance model interpretability, nomograms were developed based on the logistic regression coefficients. Each nomogram visually displays the weight of individual features on the infection risk, allowing clinicians to estimate the likelihood of infection based on a combination of parameters. Axes were scaled according to the log-odds of each predictor, with a ranked display of variables derived from the IG feature relevance scores. This provides a clinically intuitive interface for integrating complex multivariable data into actionable infection risk assessments.

## 3. Results

The characteristics of the total study cohort (Table 1) describe the 82 KT patients, of whom 56.1% were male with a mean age of 52 ± 11 years at transplantation. Seventy-two patients (87.8%) had detectable TTV before KT. At the time of cutoff analysis, the time elapsed since KT was 20.2 ± 10.3 months.

Within the 6-month outcome window, 25/82 (30.5%) patients experienced clinically significant infections, predominantly viral [17/25 (68%)] with the remainder bacterial [8/25 (32%)]; no proven invasive fungal infections were documented. For the primary breakdown, mixed viral–bacterial episodes were classified as viral. A patient-level listing (pathogen/syndrome and onset relative to sampling) is provided in Supplementary Table S1. TTV loads increased from 3.35 ± 1.67 log_10_ cp/mL before KT to 4.53 ± 1.93 log_10_ cp/mL at the time of cutoff analysis.

Biopsy-proven acute rejection within the 6-month window was rare (n = 2), precluding meaningful modeling of TTV as a bidirectional marker of over- versus under-immunosuppression in this dataset.

Additionally, 63.4% of patients received induction therapy with thymoglobulin; maintenance immunosuppression consisted of a regimen including mTOR inhibitors, calcineurin inhibitors, and prednisolone in 31.7% of patients, and tacrolimus, mycophenolic acid (MPA), and prednisolone in 68.3%; 26.8% had preformed donor-specific antibodies (DSAs); 19.5% experienced delayed graft function; and 30.5% had diabetes at the time of cohort analysis. The mean tacrolimus trough level was 6.63 ± 2.55 ng/mL, and the mean everolimus level was 5.62 ± 1.63 ng/mL.

The univariable analysis (Table 2) comparing KT patients with and without infection, revealed significantly higher TTV loads in infected patients (5.39 ± 1.68 log_10_ vs. 4.16 ± 1.94 log_10_ cp/mL, *p* = 0.0057). Infected patients were also significantly older at the time of cutoff analysis (58.5 ± 10.1 years vs. 52.0 ± 10.9 years, *p* = 0.0167), had shorter times since KT (14.7 ± 10.0 months vs. 22.6 ± 9.6 months, *p* = 0.0014), and exhibited lower eGFR values (45.0 ± 11.3 vs. 63.5 ± 19.0 mL/min/1.73 m^2^, *p* < 0.0001). No significant associations were found for gender, race, delayed graft function, or immunosuppressive therapies.

**Figure 1:**
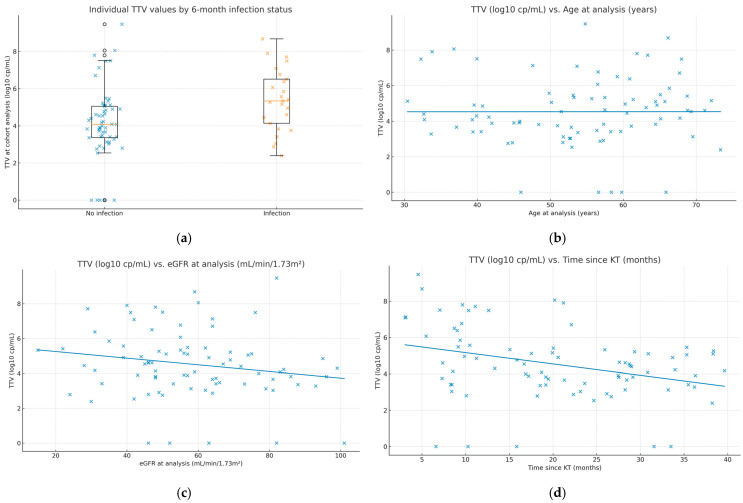
Individual TTV values and clinical correlates. (**a**) Dot/box plot of TTV at the analysis time point (log_10_ copies/mL) by 6-month infection outcome (“Infection” vs. “No infection”). Boxes show the interquartile range (IQR), center lines indicate medians, whiskers extend to 1.5× IQR, and all individual observations are overlaid. (**b**) Scatterplot of TTV vs. age at sampling with an ordinary least-squares fit; Spearman ρ = 0.104, *p* = 0.35. (**c**) Scatterplot of TTV vs. eGFR at sampling (mL/min/1.73 m^2^) with an ordinary least-squares fit; Spearman ρ = −0.201, *p* = 0.07. (**d**) Scatterplot of TTV vs. time since kidney transplantation (months) with an ordinary least-squares fit; Spearman ρ = −0.305, *p* = 0.005. Each dot represents one patient (N = 82). Infection outcomes refer to events occurring within 6 months after the sampling date.

To visualize between-patient variability and clinical correlates, we added dot/box plots of individual TTV values by subsequent infection status (Figure 1a) and scatterplots relating TTV to age, eGFR, and time since transplantation (Figure 1b–d). These displays complement the summary statistics and facilitate clinical interpretation.

To evaluate the predictive performance of TTV for detecting infections, the AUC and Youden’s index were used to determine the optimal cutoff thresholds. For TTV loads before KT, the AUC was 0.47, indicating a poor discriminatory capacity, with an optimal threshold of 5.46 log_10_ cp/mL yielding a sensitivity of 12% and a specificity of 95%.

Using ROC analysis, the Youden-optimal cutoff was 5.16 log_10_ cp/mL, yielding a sensitivity of 60% (15/25; 95% CI, 40.7–76.6) and specificity of 80.7% (46/57; 95% CI, 68.7–88.9); the AUC was 0.693 (95% CI, 0.562–0.823). Pre-transplant TTV showed poor discrimination (AUC 0.47), and the TTV ratio performed poorly (AUC 0.38).

In the reviewer-requested time windows, discrimination peaked in the mid-term interval. For <12 months (n = 26; 15 infected), the AUC was 0.51 (95% CI, 0.28–0.74); at the global 5.16 log_10_ cp/mL cutoff, sensitivity was 0.60 and specificity 0.45. For 12–24 months (n = 25; 6 infected), the AUC was 0.78 (95% CI, 0.54–1.00), with a sensitivity of 0.83 and specificity of 0.90 at the same cutoff. For >24 months (n = 31; 4 infected), the AUC was 0.51 (95% CI, 0.20–0.82), with a sensitivity of 0.25 and specificity of 0.89 at 5.16 log_10_ cp/mL. These exploratory results, limited by small stratum sizes, suggest that TTV is most informative at around 12–24 months after KT. In complementary cumulative, time-anchored analyses, performance was variable in small strata: at ≥18 months, the AUC was 0.62 with a sensitivity of 50% and specificity of 87.2% at the 5.16 log_10_ copies/mL cutoff; at ≥24 months, the AUC was 0.51 with a sensitivity of 25% and specificity of 88.9%; at ≥30 months, the AUC was 0.54 with a sensitivity of 50% and specificity of 92.9%; and at ≥36 months, the AUC was 0.50 with a sensitivity of 50% and specificity of 100%. Although precision is limited by the small number of events in later anchors, specificity remained high at the global cutoff, supporting TTV as a supportive risk marker while underscoring the need for time-specific validation in larger cohorts.

The performance of TTV across categorical windows and cumulative time anchors is summarized in Table 3, including AUC (95% CI), Youden thresholds, and operating characteristics at the global 5.16 log_10_ copies/mL cutoff.

**Figure 2:**
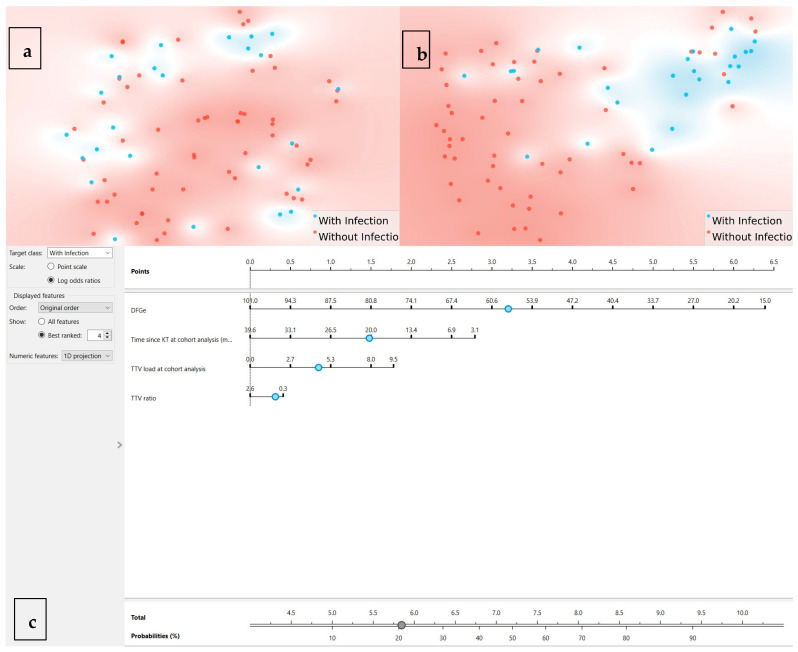
t-SNE projections and nomogram. (**a**) t-SNE using all features: each dot is one patient; blue = infection within 6 months; red = no infection; limited separation. (**b**) t-SNE using the top four features by information gain (eGFR, time since KT, TTV at analysis, TTV ratio), showing improved—but incomplete—separation. Axes are unitless and represent 2D embeddings of high-dimensional similarity; proximity indicates multivariate similarity, not causal structure. (**c**) Nomogram from the final logistic regression model: assign points per predictor, sum, and map to predicted 6-month infection probability; intended as a supportive visualization rather than a stand-alone decision tool.

As these results were somewhat disappointing, an LR was developed in Orange Data Mining to predict infection outcomes. The analysis incorporated all available features, with performance evaluated through multiple metrics. The final model achieved an AUC of 0.700, classification accuracy (CA) of 0.695, sensitivity of 0.695, and specificity of 0.552, reporting 25 misclassified cases out of 82 cases. To better understand the distribution of features and outcomes, a t-SNE visualization plot (Figure 2a) was generated. The plot highlights no clear separation between with infection (in blue) and without infection (in red) groups, indicating areas of overlap where the model struggled to differentiate between the two classes.

To further enhance the LR model’s performance and interpretability, a feature extraction analysis was conducted using information gain. The results identified four features as the most informative features (e.g., eGFR, time since KT at cohort analysis (months), TTV load at time of cutoff analysis, and TTV ratio). By incorporating the most relevant features, the refined model achieved a notable improvement in performance. The updated LR model reported an AUC of 0.797, a CA of 0.805, and an F1-score of 0.805, demonstrating a stronger predictive capacity, with sensitivity and specificity values also improved to 0.805 and 0.735, respectively. The resulting confusion matrix showed improved classification, correctly identifying 49 non-infected cases and 17 infected cases, with a reduction in misclassifications to 16 cases.

The t-SNE plot (Figure 2b) was regenerated using the refined features, revealing a clearer separation between infected and non-infected cases compared to the initial analysis. While some overlap persisted, the updated visualization demonstrated improved clustering, particularly for the infected group, suggesting that the selected features enhanced the model’s ability to distinguish between outcomes.

To further enhance the interpretability and clinical applicability of the LR model, a nomogram was created (Figure 2c). The nomogram visually represents the contribution of each feature to the probability of infection, allowing for an individualized risk assessment and supporting more nuanced immunosuppression management decisions. The most influential feature, eGFR, contributed the largest point range, followed by time since KT at the time of cutoff analysis (months), TTV load at the time of cutoff analysis, and TTV ratio. By summing the points assigned to each predictor, the total score can be converted into a probability of infection, providing a user-friendly tool for decision-making in clinical practice.

To enhance the interpretability and reproducibility of the final logistic regression model, we extracted the coefficients used in the predictive formula, which are summarized in Table 4. Each coefficient represents the strength and direction of the association between the corresponding variable and the likelihood of developing infection post-transplant. Positive coefficients suggest increased risk, while negative coefficients indicate protective effects. All continuous features were standardized (mean = 0, SD = 1) prior to model training.

The logistic regression equation used to estimate infection probability is given by

p = exp(β_0_ + β_1×1_ + β_2_X_2_ + …)/(1 + exp(β_0_ + β_1_X_1_ + β_2_X_2_ + …))

where β_0_ is the intercept and X_1_, X_2_, etc., are the standardized predictor variables.

These coefficients make the model fully transparent and reproducible, allowing future researchers and clinicians to apply the prediction formula in new patient cohorts. Although this analysis was performed in a relatively small, retrospective dataset, the structured approach adopted, combining rigorous cross-validation, feature standardization, and nomogram-based interpretation, sets the stage for prospective validation and broader implementation in clinical transplant care.

## 4. Discussion

This study explored the prognostic utility of TTV viremia in predicting infection risk in a cohort of 82 prevalent KT patients, integrating both conventional statistical and ML approaches. Our findings indicate that while the TTV viral load, particularly when measured post-transplantation, has a moderate association with subsequent infection, its predictive performance remains limited for routine clinical application in its current form.

To the best of our knowledge, this is the first study to apply ML models to predict TTV viremia as a marker of infection in KT recipients. The integration of advanced analytics enhances predictive capacity, offering a novel approach to identifying patients at a higher risk of infection.

We observed a high incidence of infections within six months following the determination of TTV levels, with 30.5% of KT recipients experiencing infectious events between the 3rd and 40th months after KT. Infection represents a major complication following KT [1]. Therefore, the implementation of immune monitoring strategies may help minimize the risk of adverse events related to over-immunosuppression, without compromising graft outcomes.

This work provides additional insight into the prevalence of TTV among patients with end-stage renal disease. In this study, 87.8% of patients had detectable TTV DNA at the time of KT. The prevalence of detectable TTV viremia in patients on hemodialysis has been estimated to range between 41.7% and 80.0% [4], higher than in healthy individuals, supporting the hypothesis that TTV replication is enhanced by the impaired immune competence associated with end-stage renal disease, as well as by a persistent low-grade inflammatory state [22,23]. Additionally, in this cohort, the median pre-transplant TTV DNA level was 3.35 log_10_ cp/mL, which is consistent with previously published data, where TTV DNA loads have consistently been reported in the range of 2.9 to 4.4 log_10_ cp/mL [4,24].

It is already known that peripheral blood TTV copy numbers are associated with the function of the host’s immune system [4]. We observed a significant increase in TTV viremia from pre-transplant levels (3.35 ± 1.67 log_10_ cp/mL) to the time of cohort analysis (4.53 ± 1.93 log_10_ cp/mL), reflecting the expected viral replication in the context of immunosuppression. This rise is consistent with previous studies that have demonstrated increased TTV replication following transplantation [25], further supporting the role of TTV as a marker of immunosuppression. Persistent TTV replication in immunocompromised patients highlights its potential utility for the longitudinal monitoring of immune function.

Sufficient data exist to support a linear, robust, and independent association between TTV load and all types of infectious events in KT recipients, including common post-transplant pathogens such as opportunistic infections, CMV, BKV, and bacterial infections [26]. The TTVguideIT study [26], a multicenter, patient- and assessor-blinded, non-inferiority, randomized controlled phase II trial, aimed to compare standard versus TTV-guided immunosuppression in kidney transplant recipients during the first year after transplantation. In the randomized TTVguideIT trial, an externally defined target range of approximately 4.6–6.2 log_10_ copies/mL was proposed for the first post-transplant year. In our cohort, TTV loads at the analysis time point were higher among patients who subsequently developed infection than among those who did not (5.39 ± 1.68 vs. 4.16 ± 1.94 log_10_ copies/mL; *p* = 0.0057). Importantly, our cohort-specific decision threshold (5.16 log_10_ copies/mL), selected by ROC analysis using Youden’s index, differs from externally proposed ranges and should be interpreted within the distinct follow-up window and case mix of this study. In our data, the 5.16 log_10_ copies/mL cutoff was selected empirically by Youden’s index to balance sensitivity and specificity. Given time- and cohort-dependence, externally proposed ranges should not be conflated with our data-driven threshold.

This inconsistency likely stems from differences in study design and patient populations. While the TTVguideIT trial and similar studies focused exclusively on kidney transplant recipients during the first post-transplant year, our cohort included prevalent transplant patients ranging from 3 to 40 months post-transplantation. As a result, the TTV viral load thresholds established in those earlier studies may not be directly applicable to our more heterogeneous and longer post-transplant population. These findings underscore the need for further research to establish TTV viral load cutoffs that are tailored to different post-transplant time points and patient characteristics.

Notably, the optimal cutoff value for the TTV load that best discriminates between patients with and without infection was 5.16 log_10_ cp/mL at the time of cutoff analysis, corresponding to a moderate sensitivity of 60% and a high specificity of 81%. With an AUC of 0.69, this cutoff value demonstrated a moderate predictive performance for infection risk. This threshold aligns with other studies suggesting a TTV cutoff between 4.5 and 5.5 log_10_ cp/mL for infection risk stratification [27,28]. A sensitivity of 60% implies that a substantial proportion of infected patients may remain undetected, potentially delaying diagnosis and management. Conversely, the 81% specificity indicates that some patients may be incorrectly identified as high-risk, possibly leading to unnecessary reductions in immunosuppressive therapy and increased risk of rejection. These clinical implications underscore the limitations of using a single cutoff value in isolation. While this threshold demonstrated moderate discriminative capacity in our cohort, it should not be used as a standalone marker. We advocate for the integration of TTV monitoring with additional clinical parameters and biomarker data to enhance infection risk stratification. The high specificity (81%) of our TTV threshold suggests its utility in confirming the infection risk when values exceed 5.16 log_10_ cp/mL, although the moderate sensitivity (60%) limits its value for ruling out infection when below this threshold. Even with the acknowledged limitations in sensitivity, our findings offer clinically relevant insights. This could support clinical decision-making regarding infection prophylaxis strategies or immunosuppression adjustment in high-risk patients, although decisions should not be based on TTV levels alone until further validation studies are completed.

Additionally, stratification of TTV loads by follow-up intervals showed a progressive improvement in the model’s predictive performance. At 36 months, the sensitivity and specificity of the threshold (5.16 log_10_ cp/mL) reached 60.0% and 76.2%, respectively. This finding underscores the utility of TTV monitoring as a long-term biomarker for infection risk and reinforces the unmet need for clinical trials designed to evaluate the role of TTV in predicting infectious risk after the first year of KT.

Because infection risk and TTV kinetics evolve post-transplant, we modeled the time since transplantation and evaluated reviewer-requested strata. Although precision is limited by the sample size, discrimination was strongest at 12–24 months and weaker earlier and later, which is biologically plausible as induction effects taper and maintenance regimens stabilize. Future studies should validate window-specific thresholds.

Nevertheless, the accuracy and clinical utility of TTV as a biomarker depend heavily on the standardization of its measurement and interpretation. Despite some efforts, this is still one of the major challenges in the field. In fact, the absence of standardized protocols for TTV quantification, including assay selection, reporting thresholds, and timing of measurement relative to transplantation, has made the acceptance of these assays in transplantation difficult. This heterogeneity complicates direct comparison across studies and may contribute to variability in reported associations between TTV levels and clinical outcomes. Additionally, while commercial assays such as the one used in this study offer broad genotype coverage and reproducibility, inter-laboratory differences in sample handling, extraction efficiency, and calibration can still impact results.

To address these issues, future studies should prioritize the development and adoption of consensus guidelines for TTV measurement and reporting. This would enhance reproducibility, facilitate meta-analyses, and accelerate the translation of TTV monitoring into routine clinical practice. Until such standards are established, careful documentation of assay methods and transparent reporting of results, as implemented in this study, remain essential to advancing the field.

The TTV copy number is directly associated with factors that determine the immune function, including age, gender, and the type and dosage of immunosuppressive drugs [26]. In this study, subgroup analyses revealed that an older age, lower eGFR, and shorter time since transplantation were associated with a higher risk of infection. These findings suggest that TTV viremia could be particularly useful for infection risk stratification in these vulnerable subgroups, offering an opportunity for closer monitoring and tailored adjustments to immunosuppressive therapy.

Several potential confounders that may influence TTV levels and the infection risk were not accounted for in our study, including nutritional status, serum albumin levels, and co-infections with other viruses. The nutritional status and hypoalbuminemia have previously been linked to the immune function and infection susceptibility in transplant recipients. Additionally, co-infections with other viruses can modulate immune responses and viral replication dynamics, potentially affecting TTV load measurements [29]. The absence of adjustment for these variables may limit the accuracy of our predictive model and should be considered when interpreting our findings.

Considering the presumed role of TTV as a surrogate marker of immune status, viral replication kinetics during the post-transplant period are primarily influenced by the amount and type of immunosuppression. Induction therapy with lymphocyte-depleting agents has been associated with higher TTV viral loads [27,30]. We were unable to observe such an association in our cohort. This discrepancy may be attributed to cohort heterogeneity—particularly the inclusion of patients at different time points post-transplantation—as well as a relatively limited sample size, which may have precluded the detection of subtler associations.

The application of ML further enhanced the predictive capacity of our model. The LR model incorporating selected features, including eGFR and time since KT, achieved an improved AUC of 0.797, with a classification accuracy of 0.805. The reduction in misclassified cases and the refined t-SNE plot suggest that integrating clinical variables with TTV loads may optimize infection risk prediction. ML approaches have increasingly demonstrated their utility in refining predictive models, particularly in transplant medicine, where complex interactions influence clinical outcomes.

Based on the LR model applied in this study, we observed a significant improvement in the performance metrics (AUC = 0.797; sensitivity = 80.5%; specificity = 73.5%), but in spite of that, a considerable number of misclassified cases remained. These misclassifications raise concerns about false positives and false negatives, which, in clinical practice, could lead to inappropriate modulation of immunosuppressive therapy. False positives may lead to unnecessary reductions in immunosuppression, increasing the risk of rejection, while false negatives could result in missed infection risks. This can be justified by the number of subjects enrolled in this study, which might be overcome with future studies with larger datasets, helping to further optimize model calibration and reduce misclassification rates.

From a clinical perspective, the integration of TTV monitoring with machine learning algorithms may support more individualized immunosuppressive strategies. In patients with persistent TTV viremia and other risk factors, early interventions such as infection screening or immunosuppression adjustments could be implemented. Conversely, patients with stable low TTV loads may benefit from immunosuppression minimization strategies, reducing long-term toxicity. However, it is important to underscore that the current model offers supportive guidance rather than enabling fully personalized treatment decisions. Its moderate predictive performance suggests that it should be used in conjunction with clinical judgment and not as a standalone tool.

A key strength of this study is the incorporation of a machine learning approach, allowing for a more nuanced analysis of infection risk compared to conventional threshold-based methods. While traditional analysis identified a TTV threshold of 5.16 log_10_ cp/mL with only a moderate ability to identify patients at risk, the ML model improved the predictive performance. By leveraging LR with LOOCV, we maximized data use and minimized overfitting. This demonstrates that even with a limited sample size, advanced analytical techniques can extract relevant clinical insights. The nomogram developed may aid clinicians in interpreting TTV values alongside other clinical variables, offering additional—but not definitive—support for risk stratification. Importantly, external validation in independent cohorts is required to confirm both the generalizability and the practical clinical utility of the model.

### 4.1. Strengths and Limitations

This study provides one of the few retrospective analyses evaluating TTV as a biomarker for infection risk in kidney transplant recipients, incorporating both pre-transplant and follow-up TTV measurements in a real-world clinical setting. The integration of an ML approach, specifically the LR model with LOOCV, enabled a robust performance assessment despite the limited sample size. The resulting nomogram offers clinicians a practical tool to estimate infection risk by combining the TTV viral load with other clinical variables, potentially advancing personalized immunosuppression management. This study was not powered for subgroup validation by age, sex, diabetes, or comorbidity strata; such analyses remain exploratory and require multicenter external validation to judge generalizability.

Furthermore, several limitations must be acknowledged. First, this study’s monocentric design and small cohort size may limit the generalizability of our findings and reduce the statistical power, particularly for subgroup analyses. Second, while the ML model improved predictive accuracy and provided individualized risk estimates, its application is limited by the modest sample size and single-center design. Despite the methodological robustness of the LOOCV approach applied to a limited dataset, this strategy does not replace true external validation. The absence of an independent test cohort is a significant limitation and restricts the assessment of the model’s generalizability. Furthermore, the small sample size and single-center design further limit the extrapolation of our findings to broader transplant populations. Future studies should prioritize external validation using independent, multicenter cohorts to confirm predictive accuracy and ensure clinical applicability. Such efforts would allow the refinement of threshold values and modeling strategies, ultimately supporting the clinical implementation of TTV-based infection risk stratification. Nonetheless, this study demonstrates the feasibility and added value of ML approaches in transplant biomarker research. Third, the wide range in timing of TTV measurement, spanning from 3 to 40 months post-KT, may obscure dynamic patterns of TTV behavior and infection risk that are known to evolve throughout the post-transplant course. By analyzing a cross-sectional cohort at varied time points, we may have introduced bias in estimating the association between TTV levels and infectious outcomes. While we attempted to account for this variability through stratified analyses by time intervals, we acknowledge that a more standardized timepoint for sampling would improve the comparability and interpretability. Fourth, the lack of association between TTV levels and different immunosuppression regimens may reflect either true biological independence or insufficient power to detect such relationships in a heterogeneous population. Fifth, although standardized commercial assays were used for TTV quantification, the absence of universally accepted protocols for TTV measurement and reporting may affect reproducibility across centers. Sixth, the lack of differentiation among infection types may affect the specificity and predictive utility of TTV as a biomarker, potentially limiting the applicability of our findings across different infection profiles. Future studies should consider detailed infection classification to better evaluate the relationship between TTV levels and specific types of infections.

Finally, the retrospective nature of infection ascertainment and the relatively short follow-up period may have led to under-detection of infectious events. Moreover, several potential confounders were incompletely captured, notably nutritional status, serum albumin, lymphocyte subsets, and co-infections beyond routine CMV/BKPyV/JCPyV surveillance, which can modulate immune competence and TTV replication; these omissions are acknowledged limitations and priorities for future data collection.

### 4.2. Clinical Implications for Personalized Immunosuppression

The potential of TTV monitoring to inform long-term immunosuppressive strategies becomes evident when combined with ML-based risk modeling. Our findings indicate that persistently elevated TTV loads coupled with certain identified factors can identify patients at a heightened infection risk. Such insights could enable clinicians to tailor immunosuppressive regimens more precisely, either by initiating early infection surveillance or cautiously reducing drug dosages to avoid over-immunosuppression.

Conversely, stable, low TTV levels may suggest adequate immune reconstitution, allowing for more aggressive immunosuppression tapering and diminished reliance on prophylactic antimicrobials. Our machine learning model, which integrated both clinical and virological variables, underscores the multifactorial nature of infection risk. As larger datasets become available and TTV assay standardization advances, the predictive accuracy of these algorithms should improve. Overall, these findings support a personalized approach to immunosuppression, balancing the need to prevent rejection with minimizing infectious complications.

While this study has demonstrated the potential of TTV viremia as a biomarker, it is essential to acknowledge its limitations, as previously presented.

Nevertheless, this study contributes novel insights by incorporating ML and long-term follow-up data (up to 40 months), extending prior work that largely focused on early post-transplant periods. The finding that TTV levels retain moderate predictive value even in prevalent KT populations supports their potential as a non-invasive, adjunct biomarker. Importantly, we also observed that factors such as older age, lower eGFR, and a shorter time since KT were independently associated with an increased infection risk, reinforcing the multifactorial nature of post-transplant vulnerability.

## 5. Conclusions

In conclusion, this study demonstrates that TTV viremia may assist in assessing the infection risk in KT recipients, with a threshold of 5.16 log_10_ cp/mL offering high specificity. Although the sample size was limited and there is a need for improved sensitivity, our ML approach represents a methodological advance in biomarker development for KT recipients. TTV monitoring, supported by machine learning, enables more personalized risk assessments and immunosuppression strategies. Although larger, multicenter studies are still required to validate this approach, our current findings establish a solid foundation and underscore the promise of viral biomarkers for truly personalized care.

## Figures and Tables

**Table 1:** Clinical and demographic data of the study population.

Characteristics of the Total Study Cohort	Total Cohort (n = 82)
Gender (male), n/%	46/56.10
Race (Caucasian), n/%	67/87.71
Age at KT (years), mean ± SD	52 ± 11
Age at time of cutoff analysis, mean ± SD	54 ± 11
Time on dialysis pre-KT (n = 81) (months), mean ± SD	158.20 ± 314.20
Type of dialysis pre-KT (hemodialysis), n/%	67/87.71
TTV load before KT (log_10_ cp/mL), mean ± SD	3.35 ± 1.67
TTV load at time of cutoff analysis (log_10_ cp/mL), mean ± SD	4.53 ± 1.93
Time elapsed since KT at time of cutoff analysis (months), mean ± SD	20.21 ± 10.29
Infections until 6 months after KT time of cutoff analysis (n)	25
Viral	17
Bacterial	8
Time elapsed between TTV analysis and infection (months), mean ± SD	3 ± 2
Biopsy-proven rejection until 6 months after KT time of cutoff analysis (n)	2
Previous KT, n/%	7/8.54
Maintenance IMS (mTOR+ calcineurinic), n/%	26/31.71
Induction IMS (thymoglobulin), n/%	52/63.41
Preformed DSAs at KT, n/%	22/26.83
Delayed graft function, n/%	16/19.51
Diabetes at time of cutoff analysis, n/%	25/30.49
Type of KT (deceased donor), n/%	71/86.59
Cold ischemia time (N = 71) (hours), mean ± SD	11.85 ± 6.75
HLA mismatches (DR)	
0	16
1	47
2	19
HLA mismatches (A and B)	
0	10
1	21
2	19
3	25
4	7
HIV +, n/%	4
Tacrolimus serum concentration (ng/mL) (n = 81), mean ± SD	6.63 ± 2.55
Everolimus serum concentration at time of cutoff analysis (ng/mL) (n = 25), mean ± SD	5.62 ± 1.63
Cause of end-stage renal disease	
Cystic kidney disease	10
Glomerular disease	27
Undefined cause	14
Hypertension	10
Diabetes	6
Chronic interstitial nephritis	3
Hereditary	5
Other	7

KT: kidney transplant; IMS: immunosuppression; DSA: donor-specific antibodies.

**Table 2:** Clinical and laboratory parameters in patients with infections and patients without infections (n = 82).

Variable	Infection (n = 25)	Without Infection (n = 57)	*p*-Value
TTV load before KT (log_10_ cp/mL)	3.22 (1.56)	3.40 (1.74)	0.653659 ^a^
TTV load at the time of cutoff analysis (log_10_ cp/mL)	5.39 (1.68)	4.16 (1.94)	0.005687 ^a^
TTV ratio (TTV load before KT/TTV load at the time of cutoff analysis)	0.76 (0.32)	0.90 (0.37)	0.123056 ^a^
Time of KT at the time of cutoff analysis (months)	14.70 (10.00)	22.63 (9.62)	0.001408 ^a^
Age at the time of cutoff analysis (years)	58.48 (10.07)	52.01 (10.99)	0.016745 ^a^
eGFR (mL/min/1.73 m^2^)	45.04 (11.29)	63.49 (18.96)	0.0000144 ^a^
Gender (male)	52.0%	57.9%	0.7999 ^b^
Race (Caucasian)	92.0%	77.2%	0.198314 ^b^
Maintenance IMS (mTOR+ calcineurin inhibitor)	No (68.0%)	No (68.4%)	1 ^b^
DSAs	No (68.0%)	No (75.4%)	0.667802 ^b^
Delayed graft function	No (72.0%)	No (84.2%)	0.326203 ^b^
Diabetes at the time of cutoff analysis	No (72.0%)	No (68.4%)	0.949331 ^b^
Induction IMS (thymoglobulin)	68.0%	61.4%	0.747533 ^b^
Type of dialysis (hemodialysis)	HD (68.0%)	HD (80.7%)	0.320709 ^b^

KT: kidney transplant; IMS: immunosuppression; DSA: donor-specific antibodies; eGFR: estimated glomerular filtration rate; a: Mann–Whitney U; b: Chi-squared.

**Table 3:** Performance of TTV for 6-month infection prediction across time windows. Summary of discrimination (AUC with 95% CI), thresholds (Youden), and operating characteristics at the global cutoff of 5.16 log_10_ copies/mL across categorical windows and cumulative time anchors. Wilson score intervals are used for sensitivities and specificities; Hanley–McNeil intervals for AUC.

Window	N	Infected	Non-Infected	AUC	AUC 95% CI	Cutoff @Youden (log_10_)	Sensitivity @Youden	Specificity @Youden	Sensitivity @5.16	Specificity @5.16
Overall (All patients)										
All patient	82	25	57	0.69	0.56–0.82	5.16	60.0%	80.7%	60.0%	80.7%
Categorical windows (<12, 12–24, >24 months)										
<12 months	26	15	11	0.51	0.28–0.74	3.04	100.0%	27.3%	60.0%	45.5%
12–24 months	25	6	19	0.78	0.54–1.00	5.16	83.3%	89.5%	83.3%	89.5%
>24 months	31	4	27	0.51	0.20–0.82	5.27	25.0%	92.6%	25.0%	88.9%
Anchored cumulative windows (≥18, ≥24, ≥30, ≥36 months)										
≥18 months	47	8	39	0.62	0.40–0.85	5.16	50.0%	87.2%	50.0%	87.2%
>24 months	31	4	27	0.51	0.20–0.82	5.27	25.0%	92.6%	25.0%	88.9%
≥30 months	16	2	14	0.54	0.09–0.98	5.27	50.0%	92.9%	50.0%	92.9%
≥36 months	6	2	4	0.50	0.00–1.00	5.27	50.0%	100.0%	50.0%	100.0%

AUC, area under the ROC curve; CI, confidence interval.

**Table 4:** Logistic regression coefficients of the final multivariable model used to predict infection risk.

Variable	Coefficient (β)
Intercept	3.788
eGFR	−0.075
Time of KT at the time of cutoff analysis (months)	−0.077
TTV load at the time of cutoff analysis (log_10_ cp/mL)	+0.187
TTV ratio (TTV load before KT/TTV load at the time of cutoff analysis)	−0.176

## Data Availability

The original contributions presented in this study are included in the article/Supplementary Material. Further inquiries can be directed to the corresponding author(s).

## Supplementary Materials

The following supporting information can be downloaded at https://www.mdpi.com/article/10.3390/idr17050107/s1, Table S1: Legend: pathogen/syndrome and onset relative to sampling.

## Abbreviations

TTV	Torque Teno Virus
KT	Kidney Transplantation
eGFR	Estimated Glomerular Filtration Rate
ROC	Receiver Operating Characteristic
AUC	Area Under the Curve
CIs	Confidence Intervals
CMV	Cytomegalovirus
BKPyV	BK Polyomavirus
JCPyV	JC Polyomavirus
LR	Logistic Regression
ML	Machine Learning
MPA	Mycophenolic Acid
mTOR	Mammalian Target of Rapamycin
PVAN	Polyomavirus Nephropathy
pPVAN	Presumptive Polyomavirus Nephropathy
PCR	Polymerase Chain Reaction
CKD-EPI	Chronic Kidney Disease Epidemiology Collaboration
t-SNE	t-distributed Stochastic Neighbor Embedding
IG	Information Gain
CA	Classification Accuracy
PPV	Positive Predictive Value
NPV	Negative Predictive Value
